# Protocol for generating a 3D culture of epiblast stem cells

**DOI:** 10.1016/j.xpro.2024.103347

**Published:** 2024-09-27

**Authors:** Viviane S. Rosa, Nanami Sato, Marta N. Shahbazi

**Affiliations:** 1 MRC Laboratory of Molecular Biology, Cambridge CB2 0QH, UK

**Keywords:** Developmental biology, Stem Cells, Organoids

## Abstract

Mouse gastrulation entails concomitant changes in cell fate, tissue shape, and embryo size. The use of a reproducible *in vitro* system is crucial for dissecting the mechanisms that coordinate these events. Here, we present a protocol for generating a 3D culture of epiblast stem cells (3D EpiSCs), which grow as epithelial spheroids mimicking key features of the gastrulating mouse embryonic epiblast. We describe steps for spheroid formation, growth, and passaging, followed by imaging or further downstream analyses.

For complete details on the use and execution of this protocol, please refer to Sato et al.^1^

## Highlights

• Steps to generate mouse embryonic stem cell-derived and epiblast-derived 3D EpiSCs
• Instructions for dissociating and passaging spheroids
• Guidance on plating 3D epiblast stem cells for immunostaining or other analyses

## Abstract (editor highlights)

Publisher’s note: Undertaking any experimental protocol requires adherence to local institutional guidelines for laboratory safety and ethics.

## Graphical abstract

**Figure:**
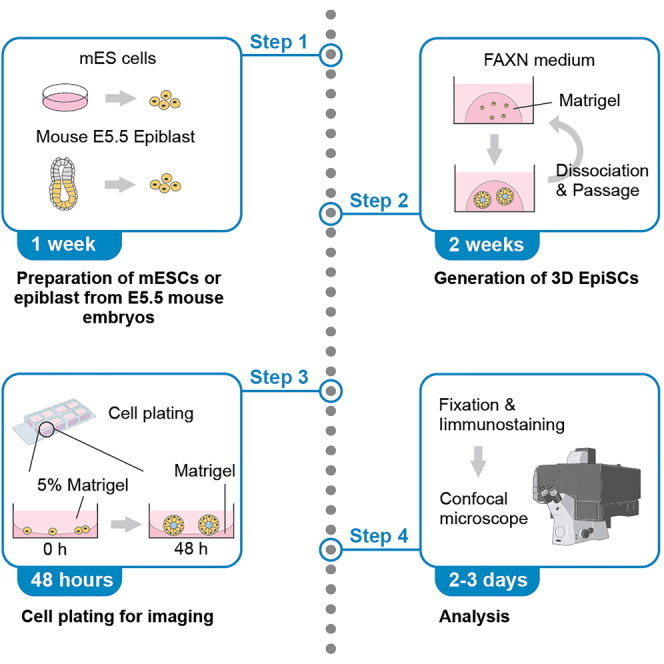

## Before you begin

The protocol below describes the specific steps for generating 3D Epiblast Stem Cells (3D EpiSCs) starting from mouse embryonic stem cells (mESCs) and mouse epiblasts at embryonic day E5.5. Although we are not describing this here, EpiSCs can also be used as a starting point to generate 3D EpiSCs, following the same steps described for mESCs, as previously reported in.^1^ Our 3D culture is a self-renewing system of 3D EpiSCs that allows genetic manipulations that would be technically challenging *in vivo*.

### Institutional permissions

3D EpiSCs can be generated from mouse embryos. In this case, it is essential to receive ethical, institutional and governmental approval before starting any experiment. All experiments involving mice performed in the UK were carried out in a UK Home Office designated facility following national and international guidelines. Our work was reviewed by the LMB Animal Welfare and Ethical Review Body (AWERB) and the University of Cambridge AWERB.

### Preparation of mESCs prior to 3D EpiSC generation

**Timing: 20 min (for step 1)**

**Timing: 20 min (for step 2)**

This section provides a step-by-step protocol for thawing and culturing mESCs, which will be used as a starting point to generate 3D EpiSCs.

**Figure 1:**
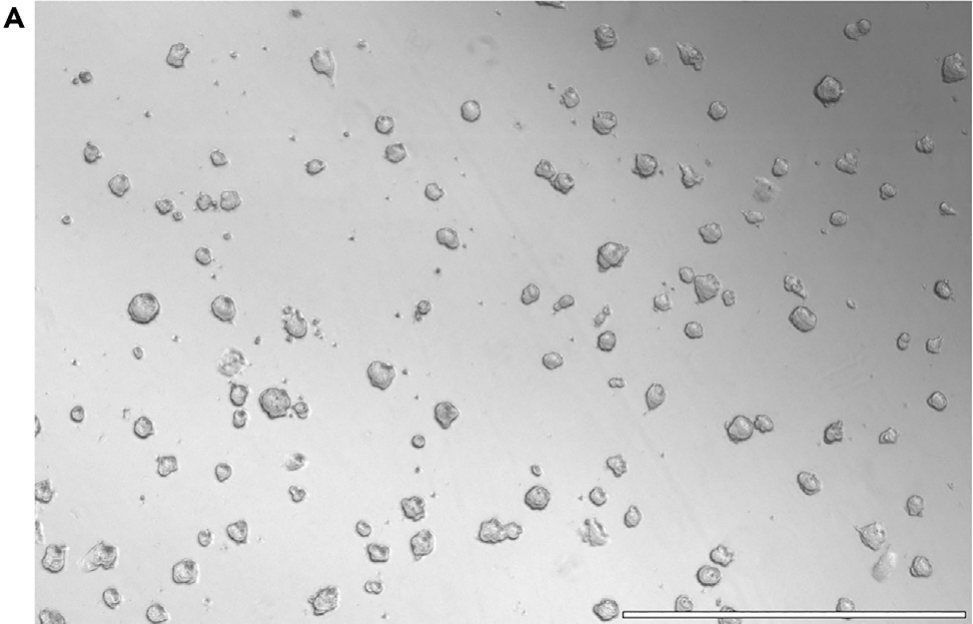
mESC culture (A) Ideal mESC density required for passaging and starting the 3D EpiSC conversion. Scale bar: 1,000 μm.

1. Thawing mESCs.
  a. Coat a well of a 12-well plate by adding 1 mL of 0.1% gelatin.
  b. Warm up a vial of mESCs at 37°C in the water bath.
  c. Add 10 mL of pre-warmed mESC base medium to a 15 mL Falcon tube. Add the mESCs immediately as soon as they are thawed.
  d. Centrifuge the cells at 200 × *g* for 5 min.
  e. After centrifugation, aspirate first the gelatin from the plate and then the supernatant from the 15 mL tube with the mESCs. Resuspend the cells in 1 mL of mESC medium (mESC base supplemented with 2iLif).
  f. Plate the cell suspension on the gelatin-coated well and place the plate in the incubator, making sure to distribute the cells evenly throughout the whole well.
  g. Replace the medium the next day after thawing with 1 mL of fresh mESC medium and every 2 days until the colonies reach the right confluence to be passaged, as shown in Figure 1A.

***Note:*** We routinely use 12-well plates for culturing mESCs, but the plate size for thawing the cells will depend on the number of cells that were frozen. The ideal density for mESCs at thawing is 2 × 10^4^ cells/cm^2^.

2. Passaging mESCs.
  a. Pre-warm mESC base medium, mESC medium and trypsin-EDTA at 20°C for 10–20 min before starting.
  b. Coat a well of a 12-well plate by adding 1 mL of 0.1% gelatin.
  c. Remove the medium from the well containing the cells and wash with 1 mL of PBS.
  d. Remove the PBS, add 500 μL of trypsin-EDTA and incubate the plate in the incubator at 37°C for 5 min.
  e. After incubation, inactivate trypsin-EDTA by adding 1 mL of mESC base medium and pipette up and down in the plate to dissociate the mESC colonies.
  f. Transfer the cell suspension to a 15 mL Falcon tube and centrifuge it at 200 × *g* for 5 min.
  g. Aspirate the gelatin from the plate.
  h. Remove the supernatant and resuspend the cell pellet in 1 mL of mESC medium. Transfer the appropriate amount/volume of cells to the gelatin-coated plate (passaging ratio 1:10–1:15). Top up to 1 mL with mESC medium.
  i. Place the plate in the incubator, making sure the cells are evenly distributed.

***Note:*** Ideally, passage mESCs every 48 h at a splitting ratio of 1:10–1:15. Splitting times and ratios might need to be adjusted depending on the mESC line used.

**CRITICAL:** Do not use overconfluent mESCs. The ideal confluency and morphology should be similar to those shown in Figure 1A.

## Key resources table

**Table:** 

REAGENT or RESOURCE	SOURCE	IDENTIFIER
**Antibodies**		

Goat pAb anti-Brachyury (dilution 1/250)	R&D Systems	Cat#AF2085; RRID:AB_2200235
Rat mAb anti-Podocalyxin (clone 192703) (dilution 1/500)	R&D Systems	Cat#MAB1556; RRID:AB_2166010
Mouse mAb anti-ZO1 (clone ZO1-1A12) (dilution 1/100)	Thermo Fisher Scientific	Cat#33-9100; RRID:AB_2533147
Rat mAb anti-β1Integrin (clone MB1.2) (dilution 1/200)	Merck	Cat#MAB1997; RRID:AB_2128202
Alexa Fluor 594 Phalloidin (dilution 1/500)	Thermo Fisher Scientific	Cat#A10239
Alexa Fluor 555 donkey anti-mouse (dilution 1/500)	Thermo Fisher Scientific	Cat#A31570; RRID:AB_2536180
Alexa Fluor 488 donkey anti-goat (dilution 1/500)	Thermo Fisher Scientific	Cat#A11055; RRID:AB_2534102
Alexa Fluor 647 donkey anti-rat (dilution 1/500)	Thermo Fisher Scientific	Cat#A48272; RRID:AB_2893138
DAPI (dilution 1/1,000)	Thermo Fisher Scientific	Cat#D3571

**Chemicals, peptides, and recombinant proteins**		

Activin-A	Qkine	Cat#Qk001
Apotransferrin	Sigma-Aldrich	Cat#T1147
B27	Thermo Fisher Scientific	Cat#10889-038
bFgf2	Qkine	Cat#Qk002
Bovine albumin fraction V	Thermo Fisher Scientific	Cat#15260037
Dispase	STEMCELL Technologies	Cat#07923
Enzyme-free cell dissociation buffer	Thermo Fisher Scientific	Cat#13151014
Fetal bovine serum	Gibco	Cat#10270-106
GlutaMAX	Thermo Fisher Scientific	Cat#35050061
Growth factor-reduced Matrigel	Corning	Cat#356231
GSK3 inhibitor CHIR99021	STEMCELL Technologies	Cat#72054
HEPES solution	Thermo Fisher Scientific	Cat#15630-056
Insulin	Sigma-Aldrich	Cat#I9287
Leukemia inhibitory factor (LIF)	Qkine	Cat#Qk018
M2 medium	Sigma	Cat#M7167
MEK inhibitor PD0325901	STEMCELL Technologies	Cat#72462
MEM non-essential amino acids	Thermo Fisher Scientific	Cat#11140035
Mouse noggin	STEMCELL Technologies	Cat#78061
Neurobasal A	Thermo Fisher Scientific	Cat#10888-022
Progesterone	Sigma-Aldrich	Cat#P8783
Putrescine dihydrochloride	Sigma-Aldrich	Cat#P5780
Rock inhibitor Y-27632	STEMCELL Technologies	Cat#72302
Sodium pyruvate	Thermo Fisher Scientific	Cat#11360070
Sodium selenite	Sigma-Aldrich	Cat#S5261
TrypLE	Gibco	Cat#12604021
0.25% trypsin-EDTA	Made in-house	N/A
XAV939	Sigma	Cat#X3004
β-mercaptoethanol	Thermo Fisher Scientific	Cat#31350-010
Tween	Sigma-Aldrich	Cat#P7949
Triton X-100	Sigma-Aldrich	Cat#101620046
Glycine	Thermo Fisher Scientific	Cat#10101620
BSA	Sigma-Aldrich	Cat#A3311
Dimethyl sulfoxide	Sigma-Aldrich	Cat#D8418-100mL
Paraformaldehyde (PFA)	Thermo Fisher Scientific	Cat#28908

**Experimental models: Cell lines**		

Mouse: E14 wild-type mESCs	Prof. Jenny Nichols, MRC Human Genetics Unit, UK	N/A
Mouse: LifeAct-GFP Brachyury IRES H2B-mCherry mESCs	Derived in-house	N/A
Mouse: C57BL/6 wild-type mESCs	Derived in-house	N/A

**Experimental models: Organisms/strains**		

Mouse: WT Hsd:ICR (CD1)	Bred in-house	N/A

**Other**		

Non-adherent multi-well plate	CELLSTAR	Cat#662102
μ-Slide 8 Well high	ibidi	Cat#80806
EVOS FL microscope	Life Technologies	Cat#AMF4300
30G needle	BD Microlance	Cat#304000
Microcentrifuge	Eppendorf	Cat#5702 R
4-well dish	Thermo Fisher Scientific	Cat#176740
35 × 10 mm tissue culture dish	Corning	Cat#353001
Cell recovery solution	Corning	Cat#354253

## Materials and equipment

**Table:** Gelatin

Reagent	Final concentration	Amount
Gelatin	0.1%	0.1 g
Milli-Q Water	N/A	100 mL
**Total**	**N/A**	**100 mL**

It can be stored at 4°C or 20°C–25°C for up to 6 months.

***Note:*** Autoclave the gelatin solution.

**Table:** mESC base medium

Reagent	Final concentration	Amount
DMEM	N/A	500 mL
Fetal Bovine Serum (FBS)	15%	90 mL
Penicillin-Streptomycin	1%	6 mL
GlutaMAX	1%	6 mL
MEM non-essential amino acids	1%	6 mL
Sodium pyruvate	1%	6 mL
β -mercaptoethanol	100 μM	1.2 mL
**Total**	**N/A**	**615.2 mL**

Store at 4°C, for up to one month.

***Note:*** Heat inactivate the FBS for 30 min at 56°C, aliquot it in 45 mL aliquots and keep it at −20°C for up to 6 months.

**Table:** mESC medium

Reagent	Final concentration	Stock concentration	Amount
ESC Base medium	N/A	N/A	10 mL
PD0325901	1 μM	10 mM	1 μL
CHIR99021	3 μM	10 mM	3 μL
Mouse Lif	10 ng/mL	10 μg/mL	10 μL
**Total**	**N/A**	**N/A**	**10 mL**

Store at 4°C for up to one week.

**Table:** 3D EpiSC base medium

Reagent	Final concentration	Amount
DMEM F12	N/A	20 mL
Neurobasal A	N/A	20 mL
N2	0.5%	200 μL
B27	1%	400 μL
GlutaMAX	1%	400 μL
Penicillin-Streptomycin	1%	400 μL
β -mercaptoethanol	100 μM	80 μL
**Total**	**N/A**	**41.48 mL**

Store at 4°C for up to one month.

**Table:** 3D EpiSC medium complete (3D FAXN)

Reagent	Final concentration	Stock concentration	Amount
3D EpiSC Base medium	N/A	N/A	10 mL
Activin A	50 ng/mL	50 μg/mL	10 μL
bFgf2	12 ng/mL	50 μg/mL	2.4 μL
Mouse Noggin	150 ng/mL	300 μg/mL	5 μL
XAV939	5 μM	5 mM	10 μL
**Total**	**N/A**	**N/A**	**10 mL**

Store at 4°C for up to one week.

**Table:** Wash Buffer

Reagent	Final concentration	Amount
DMEM F12	N/A	48 mL
Fetal Bovine Serum (FBS)	1%	500 μL
GlutaMAX	1%	500 μL
Penicillin-Streptomycin	1%	500 μL
HEPES	1%	500 μL
**Total**	**N/A**	**50 mL**

Store at 4°C for up to one month.

**Table:** N2 supplement

Reagent	Final concentration	Stock concentration	Amount
DMEM / F12	N/A	N/A	3.2 mL
Insulin	2.5 mg/mL	10 mg/mL	1.5 mL
Apo-transferrin	10 mg/mL	100 mg/mL	600 μL
BSA fraction V	0.75%	7.5%	600 μL
Progesterone	2 μg/mL	0.6 mg/mL	20 μL
Putrescine dihydrochloride	1.6 mg/mL	160 mg/mL	60 μL
Sodium selenite	0.6 μg/mL	0.6 mg/mL	6 μL
**Total**	**N/A**	**N/A**	**6 mL**

Store at −20°C for up to three months.

***Note:*** Aliquot the N2 supplement in small volumes to avoid repetitive freezing and thawing.

**Table:** Permeabilization Buffer

Reagent	Final concentration	Amount
PBS	N/A	27 mL
Triton X-100	0.3%	90 μL
Glycine	0.1 M	3 mL (Stock 1 M)
**Total**	**N/A**	**30 mL**

Store at 20°C–25°C for up to three months.

**Table:** Blocking Buffer

Reagent	Final concentration	Amount
PBS	N/A	30 mL
BSA	3%	0.9 g
Tween	0.1%	30 μL
**Total**	**N/A**	**30 mL**

Aliquot it and store it at −20°C for up to six months.

## Step-by-step method details

### Generation of 3D EpiSCs from mESCs

**Timing: 1 h**

This section provides a step-by-step protocol for the generation of 3D EpiSCs starting from mESCs.

***Note:*** After thawing the mESCs, it is important to passage the cells at least once before starting the 3D EpiSC conversion.

***Note:*** One hour before starting the mESC dissociation, place the non-adherent 24-well plate in the incubator and put Matrigel to thaw on ice. Matrigel needs to be kept on ice during the whole procedure. Using a non-adherent plate minimizes the attachment of cells in the bottom of the Matrigel drop to the plate.

1. Remove the medium from the mESC plate and wash with 1 mL of PBS.
2. Remove the PBS, add 500 μL of trypsin-EDTA and incubate it at 37°C for 5 min.
3. After incubation, inactivate the trypsin-EDTA by adding 1 mL of mESC base medium. Pipette up and down in the plate to dissociate the mESC colonies.
4. Transfer the cell suspension to a 15 mL Falcon tube and centrifuge it at 200 × *g* for 5 min.
5. After centrifugation, remove the supernatant and resuspend the cell pellet in 1 mL of mESC base medium. Pipette up and down to ensure a single-cell suspension for counting.
6. Count the cells, transfer 40,000 cells to a 1.5 mL Eppendorf tube and add 1 mL of PBS to wash the cells.

***Note:*** We recommend plating 20,000 cells per drop of Matrigel and at least 2 drops at the beginning of the conversion. The number of cells used per drop is optimized mainly for E14 WT mESCs. Different cell lines might require an increased initial cell number per drop. For example, the conversion of C57BL/6 mESCs is slower and there are high levels of cell death. In this case, we recommend doubling the initial seeding density.

7. Centrifuge the cells at 200 × *g* for 5 min.
8. Remove the PBS carefully and place the pellet on ice.

**CRITICAL:** It is important to remove as much as possible of the PBS as it can dilute the Matrigel and affect the efficiency of spheroid formation. Do not leave more than 5 μL of PBS on the pellet with the cells.

**Figure 2:**
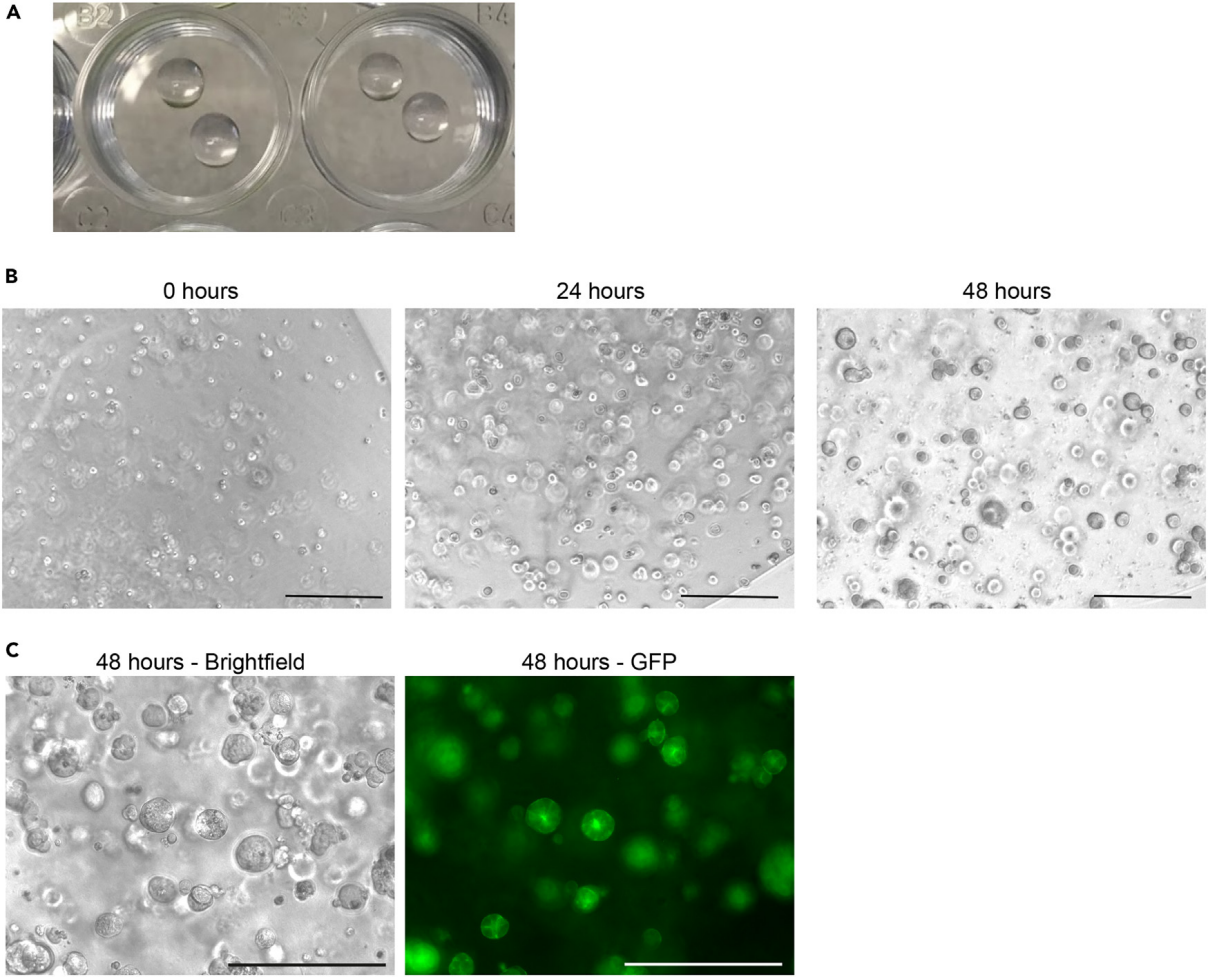
mESC-derived 3D EpiSCs (A) Matrigel drops containing 20,000 mESCs each. (B) Ideal cell density at 0, 24, and 48 h after plating. Scale bar: 250 μm. (C) LifeAct-GFP 3D EpiSCs 48 h after passaging. Scale bar: 200 μm.

9. Loosen up the pellet by rubbing the Eppendorf tube against the grid of the tissue culture hood.
10. Using cold tips, add 50 μL of 100% ice-cold Matrigel and pipet it up and down. Try to avoid bubbles.
11. Pipette 2 drops of 25 μL each in a well of a 24-well non-adherent plate as shown in Figure 2A. The ideal confluency at plating for E14 mESCs is shown in Figure 2B, 0 h.

***Note:*** The non-adherent plate should be removed from the incubator before plating the cells on it.

***Note:*** Matrigel should not touch the walls of the well. If needed, it is possible to plate one drop of Matrigel per well.

12. Place the plate in the incubator for 10 min for the Matrigel to solidify.
13. Add 600 μL of 3D FAXN medium per well.

***Note:*** Some cell lines might require the addition of Rock inhibitor in the first 24 h after the initial plating. In this case, add 10 μM of Rock inhibitor in the medium. The ideal confluency after 24 h of culture is shown in Figure 2B.

***Note:*** Ideally, passage 3D EpiSCs every 48 h or when the spheroids reach the morphology shown in Figures 2B and 2C.

***Note:*** We consider the plating day as passage 0 for 3D EpiSCs.

***Note:*** The transition from naïve (mESCs) to primed (3D EpiSCs) requires at least 4 to 5 passages. Downstream analyses should be done from passage 5 and up to passage 15 as the differentiation levels tend to increase over time in culture.^1^

**Figure 3:**
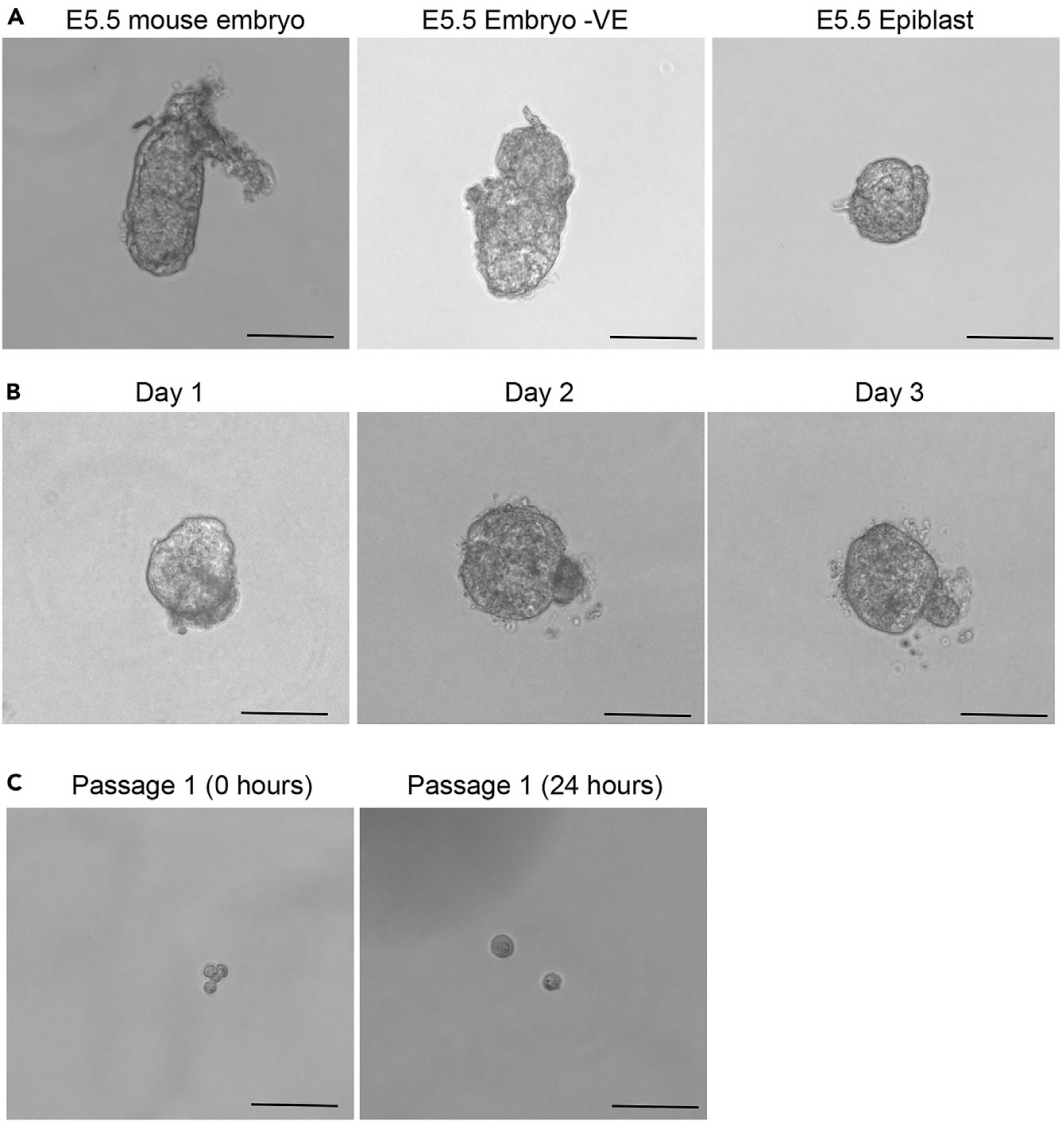
Epiblast-derived 3D EpiSCs (A) Bright-field images of an E5.5 mouse embryo. The intact embryo, the embryo devoid of visceral endoderm (VE) and the isolated epiblast are shown. Scale bars: 100 μm. (B) Bright-field images of isolated epiblasts plated in Matrigel and FAXN medium at different days. Scale bars: 100 μm. (C) Bright-field images of epiblast cells after the first passage at different time points. Scale bars: 100 μm.

### Derivation of 3D EpiSC from mouse epiblasts

**Timing: 2.5 h (for step 14)**

**Timing: 1 h (for step 15)**

14. Isolation of E5.5 epiblasts.
  This section provides a step-by-step protocol for the establishment of 3D EpiSCs from mouse epiblasts at embryonic day 5.5 (E5.5).
  ***Note:*** Before starting the epiblast dissection, place the non-adherent 24-well plate in the incubator and put the Matrigel to thaw on ice. Matrigel needs to be kept on ice during the whole procedure. Using a non-adherent plate minimizes the attachment of cells in the bottom of the Matrigel drop to the plate.
  ***Note:*** Make sure the relevant institutional, local, and national approvals are in place before starting any work with mice.
  a. Humanely cull the pregnant female by cervical dislocation and collect the uterus containing the embryos in a tube with M2 medium.
    ***Note:*** The person carrying out this procedure must have received the appropriate training beforehand.
  b. Place the uterus in a 35-mm Petri dish with M2 medium and using extra fine forceps separate the decidual tissue.
  c. Carefully open the decidua and scoop the embryo out from the implantation site.
  d. Remove manually the Reichert’s membrane using a 30 G needle while holding the embryo with the forceps from the ectoplacental cone.
  e. Prepare a 4-well dish with 500 μL of enzyme-free cell dissociation buffer in one well and 500 μL of M2 in a second well.
  f. Incubate the embryos in the enzyme-free cell dissociation buffer for 30 min at 4°C.
  g. After 30 min, move the embryos to the well that contains M2.
  h. Using a manually pulled narrow glass pipette with a diameter approximately equivalent to the short axis of the epiblast, carefully pipette in the embryos from the extra-embryonic ectoderm region. Upon pipetting out, the visceral endoderm should separate from the embryo as an entire sheet of epithelial cells. If needed, the pipetting can be repeated 2–3 more times (Figure 3A).
    ***Note:*** Using a glass pipette of the correct diameter is critical. Pull multiple pipettes beforehand and choose the most appropriate one for each embryo.
  i. Using a micro knife, manually remove the extra-embryonic ectoderm, isolating the epiblast (Figure 3A).
    ***Note:*** This same protocol can be used to isolate the epiblast for other different purposes.
  j. Make a 25 μL drop of Matrigel in a well of a non-adherent 24-well plate and place one isolated epiblast at the center of the drop.
  k. Place the plate in the incubator for 10 min for the Matrigel to solidify and add 600 μL of 3D FAXN medium per well. After 3 days in culture, the epiblasts will be ready to be passaged (Figure 3B).
15. First passage of epiblast-derived 3D EpiSCs.
  This section provides a step-by-step protocol for passaging the isolated epiblasts after 3 days of culture in 3D FAXN conditions.
  ***Note:*** Before starting, put Matrigel to thaw on ice and cool down a microcentrifuge to 4°C.
  a. Carefully remove the medium to avoid damaging the Matrigel drop and add 1 mL of dispase.
  b. Pipette wash buffer in and out of a 1 mL tip.
    ***Note:*** This step is important to prevent the isolated epiblast from attaching to the tip.
  c. Using the same tip from step b, pipette up and down carefully to break the Matrigel.
    ***Note:*** Perform this step under a magnifying scope to keep track of the epiblast and make sure it is not lost during pipetting.
  d. Incubate the plate at 37°C for 20 min.
  e. Make a drop of TryplE and a drop of wash buffer on the lid of a 35 mm Petri dish.
  f. Pipette wash buffer in and out of a 20 μL tip, and with the same tip, place the isolated epiblast in TryplE.
  g. Incubate at 37°C for 4 min.
    ***Note:*** Ideally, this step should be performed on a heated stage.
  h. Pipette wash buffer in and out of a 20 μL tip, and with the same tip, move the epiblast to the wash buffer drop.
  i. Using a very narrow manually pulled glass pipette, pipette the epiblast up and down several times until it breaks into single cells and small groups of cells (Figure 3C).
    ***Note:*** Using a glass pipette of the correct diameter is critical. Pull multiple pipettes beforehand and choose the most appropriate. The diameter should be slightly bigger than the diameter of a single cell.
  j. Make a new drop of ice-cold Matrigel using the same non-adherent 24-well plate.
  k. Manually collect the cells using the glass pipette minimizing the amount of liquid that is collected, and pipette them into the Matrigel dome.
  l. Place the plate in the incubator for 10 min to allow the Matrigel to solidify and add 600 μL of 3D FAXN medium and 10 μM of Rock inhibitor.
  m. Next day, change the medium to fresh 3D FAXN without Rock inhibitor.
  n. After 2 days clear spheroids should emerge.
    ***Note:*** After a few passages, the morphology of epiblast-derived 3D EpiSCs will become similar to the mESC-derived 3D EpiSCs.
    ***Note:*** From the second passage onwards, follow the steps described in the section below for passaging.

### Passaging 3D EpiSCs

**Timing: 1 h**

This section provides a step-by-step protocol for passaging already established mESC-derived and epiblast-derived 3D EpiSCs.

***Note:*** Before starting, place the non-adherent 24-well plate in the incubator for 1 h, put Matrigel to thaw on ice and cool down a microcentrifuge to 4°C.

16. Carefully remove the medium to avoid damaging the Matrigel drops.
17. Add 1 mL of dispase for every 2 drops and pipette up and down 10 times to break the Matrigel. Incubate the plate at 37°C for 20 min.
18. Collect the cell suspension in a 1.5 mL Eppendorf tube and centrifuge it at 300 × *g*, 4°C for 4 min.
19. Carefully remove the supernatant, add 1 mL of TrypLE, pipette it up and down 10 times and incubate at 37°C for 4 min.
20. Centrifuge the cells at 300 × *g*, 4°C for 4 min.
21. After centrifugation, carefully remove the supernatant, add 1 mL of wash buffer and pipette up and down 10 times to wash the pellet containing small clumps of cells.
22. Centrifuge the cells at 300 × *g*, 4°C for 4 min.
23. Carefully remove the supernatant and loosen up the pellet by rubbing the Eppendorf tube against the grid of the tissue culture hood. With a pipette, check the final volume of the dissolved pellet.
24. Transfer to a new tube the volume needed for plating (based on the splitting ratio and the number of drops required).

***Note:*** Ideally, passage 3D EpiSCs at a splitting ratio of 1:5. It might need to be adjusted accordingly with the growth rate of the cell line used.

25. Add the volume of 100% ice-cold Matrigel needed for the number of drops required using 25 μL per drop. For example, if you require 4 drops, add 100 μL of Matrigel, pipette up and down avoiding bubbles, and plate 25 μL per drop (maximum 2 drops per well in a 24-well plate). See Figure 2A.
26. Place the plate in the incubator for 10 min to allow the Matrigel to solidify and add 600 μL of 3D FAXN medium per well.

***Note:*** Some cell lines might require the addition of a Rock inhibitor in the first 24 h after passaging. If this is the case, add 10 μM of Rock inhibitor in the medium.

### Freezing down and thawing 3D EpiSCs

**Timing: 1 h (for step 27)**

**Timing: 30 min (for step 28)**

After 4–5 passages, 3D EpiSCs are in a primed pluripotent state and can be frozen down and thawed for further use. This section provides a step-by-step protocol for freezing down and thawing already established mESC-derived and epiblast-derived 3D EpiSCs.

27. Freezing down 3D EpiSCs.
  ***Note:*** Before starting, cool down a microcentrifuge to 4°C.
  a. To dissociate the 3D EpiSCs, follow the steps described in the section “passaging 3D EpiSCs”, from steps 16 to 22.
  b. After washing the pellet with wash buffer and centrifugating it, remove the supernatant and resuspend the cells in the freezing medium containing 500 μL of 3D FAXN medium, 400 μL of FBS and 100 μL of DMSO. Add 10 μM of Rock inhibitor in the freezing medium.
  c. Transfer the cells to a cryotube and place it in −80°C for up to one week before transferring the cryotubes to the liquid nitrogen storage container.
28. Thawing 3D EpiSCs.
  ***Note:*** Before starting, place the non-adherent 24-well plate in the incubator for 1 h and put Matrigel to thaw on ice.
  a. Place the cryotube containing the frozen 3D EpiSCs in a 37°C water bath for 1–2 min, until the cells are thawed.
  b. As soon as the cells are thawed, dilute them in 10 mL of DMEM F12 in a Falcon tube and centrifuge them at 200 × *g* for 5 min.
  c. After centrifugation, remove carefully the supernatant and place the Falcon tube on ice.
    **CRITICAL:** Do not leave more than 5 μL of DMEM F12 on the pellet with the cells as it can dilute the Matrigel and affect the efficiency of spheroid formation.
  d. Loosen up the pellet by rubbing the Falcon tube against the grid of the tissue culture hood.
  e. Using cold tips, add 50 μL of 100% ice-cold Matrigel and pipet it up and down. Try to avoid bubbles.
  f. Pipette 2 drops of 25 μL each in a well of a 24-well non-adherent plate.
    ***Note:*** The number of drops to be plated after thawing the 3D EpiSCs is the same number of drops that was frozen.
  g. Place the plate in the incubator for 10 min for the Matrigel to solidify.
  h. Add 600 μL of 3D FAXN medium per well plus 10 μM of Rock inhibitor for the first 24 h after thawing.
  i. Next day, change the medium to fresh 3D FAXN without the addition of Rock inhibitor.

### Plating 3D EpiSCs for immunostaining

**Timing: 1 h**

This section provides a step-by-step protocol for dissociating mESC-derived and epiblast-derived 3D EpiSCs and plating them for immunostaining or other downstream analyses.

***Note:*** For plating the 3D EpiSCs for immunostaining, we use a 3D on-top protocol instead of plating the cells embedded in Matrigel drops, which facilitates the staining and imaging acquisition. The 3D on top protocol consists of plating the cells on a layer of 100% Matrigel and adding medium with 5% Matrigel.^2^ This alternative protocol does not affect the efficiency of the spheroid formation.

***Note:*** We recommend using an μ-Slide 8 Well as it is compatible with 3D culture and imaging.

29. For coating an 8-well ibidi plate, using cold tips, spread 40–50 μL of 100% ice-cold Matrigel per well, avoiding bubbles and making sure the Matrigel is evenly distributed through the whole well.
30. Place the ibidi plate in the incubator for 7–10 min at 37°C to allow the Matrigel to solidify.
31. After the incubation, add 250 μL of DMEM F12 to each well pre-coated with Matrigel.

***Note:*** This step is important as it prevents the Matrigel from overdrying until the cells are ready to be plated.

32. To dissociate the 3D EpiSCs before plating for immunostaining, follow the steps described in the section “passaging 3D EpiSC”, from steps 16 to 22.
33. After washing the pellet with wash buffer and centrifugating it, remove the supernatant and resuspend the cells in 1 mL of N2B27.
34. Remove the medium from the wells on the ibidi plate and plate 250 μL of cell suspension per well, this is a 1:4 ratio.

***Note:*** The plating ratio depends on the initial confluence of the drops, it can vary from 1:4 to 1:8 ratios.

35. Place the ibidi plate in the incubator and incubate it for 10–15 min at 37°C to allow the cells to attach to the Matrigel layer.
36. In the meantime, prepare the 3D FAXN medium to be added on top of the cells by diluting 5% of Matrigel in cold 3D FAXN medium. Use 250 μL of medium per well.

**CRITICAL:** Matrigel should be dissolved in cold 3D FAXN medium using cold tips. After diluting the Matrigel in 3D FAXN medium keep it at 20°C–25°C to avoid adding cold medium to the cells.

37. After the 10–15 min incubation, remove the medium carefully from the ibidi plate and add 250 μL of the Matrigel-containing 3D FAXN medium on top of the cells.

**CRITICAL:** Add the medium carefully and close to the wall of each well to avoid the detachment of the cells from the Matrigel layer.

38. Cells can be harvested at any point for downstream analyses. For immunostaining, we provide a detailed protocol below.

### Fixation and immunostaining

**Timing: 2 days**

This section provides a standard immunostaining protocol using markers for analysis of the epithelial morphology and levels of differentiation.

***Note:*** After plating, 3D EpiSCs can be subjected to different treatments (e.g., inhibitors or growth factors). Different incubation times and stages of treatment might be required.

39. Fix the cells using 4% paraformaldehyde (PFA) for 20 min at 20°C–25°C.

***Note:*** PFA is light-sensitive. Keep the cells in the dark during the fixation.

40. Wash three times with PBS 0.1% Tween.
41. Permeabilize the cells by adding 250 μL of permeabilization buffer per well and incubate it for 30 min at 20°C–25°C.
42. Remove the permeabilization buffer and wash once with PBS 0.1% Tween.
43. Remove the PBS 0.1% Tween and add the specific primary antibodies diluted in blocking buffer (150 μL per well).

**Figure 4:**
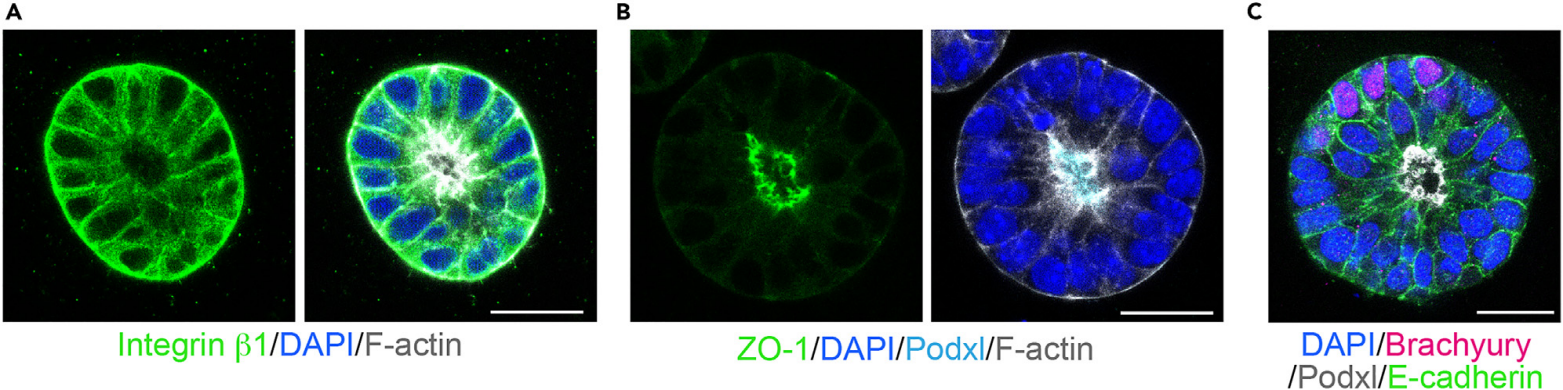
Immunostaining of 3D EpiSCs (A and B) Immunofluorescence images showing the localization of apical and basolateral markers. Scale bars: 25 μm. (C) Immunofluorescence image showing the presence of differentiated cells (Brachyury+). Scale bar: 25 μm.

***Note:*** For analyzing epithelial morphology and differentiation, use a lumen marker (e.g., Podocalyxin), a membrane marker (e.g., E-cadherin or F-actin), an apical marker (e.g., ZO-1 or aPKC), a basolateral marker (e.g., Integrin β1) and a primitive streak marker (e.g., Brachyury). See Figure 4.

44. Incubate for 24 h at 4°C.
45. The next day, wash three times with PBS 0.1% Tween and add the respective secondary antibodies with DAPI diluted in blocking buffer (150 μL per well).
46. Incubate for 2 h at 20°C–25°C and wash three times with PBS 0.1% Tween. The samples are ready to be imaged using confocal microscopy.

## Expected outcomes

This protocol describes the generation of self-renewing 3D EpiSCs from either mESCs or dissected epiblast at E5.5. In the presence of Activin-A, bFGF2, a Wnt inhibitor (XAV939), and a BMP inhibitor (Noggin), 3D epithelial spheroids with a central lumen appear within 1–2 days after plating (Figures 2B and 2C). After 2 weeks (approximately 5–7 passages), 70% of the spheroids show a single lumen, 22% have no lumen, and 8% display multiple lumens (Figure 4). At this stage, the expression of primitive streak markers is apparent in approximately 6% of the cells (Figure 4). Of these, 80% are found basally localized and not in contact with the lumen (Figure 4). 3D EpiSCs can be maintained in culture for 6 months. However, upon prolonged passaging (15 passages), an increase in differentiation is observed. In another publication it has been reported that a Bmp inhibitor is not necessary to preserve pluripotency in 3D.^3^ However, we have found that its absence increases the level of differentiation.^1^

## Limitations

The efficiency of 3D EpiSC generation is highly dependent on the mESC line (or mouse strain) that is used as a starting point. For some mESCs, it may not be possible to generate 3D EpiSCs, or these may appear at a very low efficiency. This variability across mESC lines derived from different strains affects the efficiency of 2D differentiation protocols,^4^ as well as the ability to generate stem cell-based embryo models.^5^^,^^6^ Genetic differences between different backgrounds are likely responsible of this variability.^4^ We have not observed differences in efficiency based on the passage number of mESCs or the use of genome editing techniques.

## Troubleshooting

### Problem 1

Spheroids do not form after the first passage in Matrigel and cells die (passaging 3D EpiSCs).

### Potential solution

Increase the initial density of cells that are plated in Matrigel. Combine two drops of spheroids into a single one to increase the density. Add Rock inhibition during passaging. To make sure there are no issues with the experimental protocol, we advise to include a positive control using WT E14 mESCs.

### Problem 2

Matrigel is not well dissolved during passaging (passaging 3D EpiSCs).

### Potential solution

Make sure the microcentrifuge is at 4°C.

As an alternative protocol to remove the Matrigel, BD Cell Recovery Solution can be used. This is particularly helpful if it is important to preserve protein localization and the structural integrity of the spheroids for downstream analyses (e.g., western blot or immunoprecipitation). The detailed protocol is provided below.

• Remove the culture medium and wash with PBS twice.
• Add 1 mL of cold Cell Recovery Solution to the well and pipette up and down carefully to break the Matrigel.
• Incubate the plate for 20 min on ice.
• Pipette up and down carefully, and break up the Matrigel without damaging the spheroids.
• Collect the spheroid suspension in a 1.5 mL Eppendorf tube and spin down at 300 × *g*, 4°C for 4 min. Wash with cold PBS three times to remove the Cell Recovery Solution completely.
• The pellet is ready for cell lysis.

### Problem 3

Spheroids are not properly dissociated into small groups of cells during passaging (passaging 3D EpiSCs).

### Potential solution

Make sure you are pipetting 10 times after the TrypLE incubation to break the spheroids into small aggregates.

### Problem 4

Most of the spheroids are too large and show a multi-lumen phenotype (passaging 3D EpiSCs).

### Potential solution

Reduce the number of cells at plating.

## Resource availability

### Lead contact

Further information and requests for resources and reagents should be directed to and will be fulfilled by the lead contact, Marta N. Shahbazi (mshahbazi@mrc-lmb.cam.ac.uk).

### Technical contact

Questions about the technical specifics of performing the protocol should be directed to and will be answered by the technical contact, Viviane S. Rosa (vdsrosa@mrc-lmb.cam.ac.uk).

### Materials availability

All 3D EpiSC lines generated in this study are available under a material transfer agreement.

### Data and code availability

This study did not generate datasets or analyze code.

## Acknowledgments

Work in the Shahbazi lab is supported by the 10.13039/501100000265Medical Research Council, as part of United Kingdom Research and Innovation (MC_UP_1201/24), and the 10.13039/501100000266Engineering and Physical Sciences Research Council (Horizon Europe guarantee funding, EP/X023044/1). N.S. is supported by a 10.13039/501100001691JSPS Overseas Research Fellowship, and V.S.R. is supported by a Milstein fellowship.

## Author contributions

V.S.R. developed the initial protocol, performed *in vitro* experiments, and wrote the first version of the manuscript. N.S. helped with *in vitro* experiments and manuscript writing. M.N.S. conceived the method, developed the initial protocol, performed *in vitro* and *in vivo* experiments, helped write the manuscript, provided funding, and supervised the project.

## Declaration of interests

The authors declare no competing interests.
